# BNT162b2 Third Booster Dose Significantly Increases the Humoral Response Assessed by Both RBD IgG and Neutralizing Antibodies in Renal Transplant Recipients

**DOI:** 10.3389/ti.2022.10239

**Published:** 2022-03-21

**Authors:** Tammy Hod, Aharon Ben-David, Liraz Olmer, Noa Scott, Ronen Ghinea, Eytan Mor, Itzchak Levy, Victoria Indenbaum, Yaniv Lustig, Ehud Grossman, Galia Rahav

**Affiliations:** ^1^ Renal Transplant Center, Sheba Medical Center, Tel Hashomer, Israel; ^2^ Sackler Faculty of Medicine, Tel-Aviv University, Tel-Aviv, Israel; ^3^ Bio-statistical and Bio-mathematical Unit, The Gertner Institute of Epidemiology and Health Policy Research, Sheba Medical Center, Tel Hashomer, Israel; ^4^ The Infectious Diseases Unit, Sheba Medical Center, Tel Hashomer, Israel; ^5^ Central Virology Laboratory, Public Health Services, Ministry of Health and Sheba Medical Center, Tel-Hashomer, Israel; ^6^ Internal Medicine Wing, Sheba Medical Center, Tel Hashomer, Israel

**Keywords:** immunosuppression, humoral response, renal transplantation, COVID-19 vaccine, antibody response

## Abstract

**Background:** An impaired humoral response to full dose of BNT162b2 vaccine was observed in renal transplant recipients (RTR).

**Methods:** To reveal predictors for humoral response to third vaccine, patients were stratified to positive (N = 85) and negative (N = 14) response groups based on receptor-binding domain (RBD) IgG ≥1.1 and neutralizing antibodies (NA) ≥ 16 dilution versus RBD IgG <1.1 or NA < 16, respectively. NA were detected using a SARS-CoV-2 pseudo-virus.

**Results:** Response rate increased from 32.3% (32/99) before the third dose to 85.9% (85/99) post-third vaccine with a significant rise in geometric mean titers (GMTs) for RBD IgG and NA [0.79 (95% CI 0.65–0.96) vs. 3.08 (95% CI 2.76–3.45), *p* < 0.001 and 17.46 (95% CI 12.38–24.62) vs. 362.2 (95% CI 220.7–594.6), *p* < 0.001 respective. 80.6% (54/67) seroconverted and 96.9% (31/32) remained positive following the vaccine with a significant increase in GMTs for RBD IgG and NA. Age, ESRD secondary to diabetic nephropathy (DN) and renal allograft function were independent predictors for antibody response in RTR. Mycophenolic acid (MPA) use and dose had no impact on humoral response following the third booster. AEs were recorded for 70.1% of RTR population. Systemic AEs were more common in recipients with a positive humoral response as opposed to non-responders (45.2% versus 15.4% respectively, *p* = 0.04).

**Conclusion:** 85.9% of RTR develop NA to BNT162b2 third vaccine, found effective in both negative and positive responders prior to the vaccine. Antigenic re-exposure overcame the suppressive effect of MPA on antibody response in RTR.

## Graphical Abstract

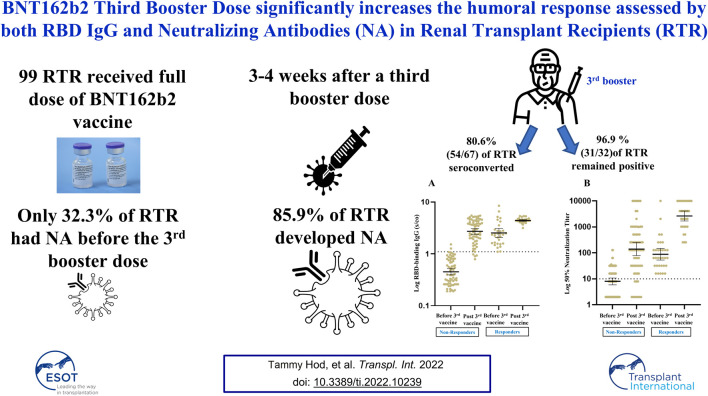

## Introduction

Renal transplant recipients (RTR) among other solid organ transplant (SOT) recipients and immunosuppressed individuals are susceptible to significant morbidity and mortality from COVID-19 infection (1). A national campaign to vaccinate this vulnerable population took place with different studies reporting impaired response to the BNT162b2 mRNA vaccine (2–5). Over 60% of RTR did not develop an adequate humoral response, with seroconversion rates being as low as 5.7% in patients receiving belatacept (6). Studies of the vaccinated RTRs showed that the main factor impairing the ability to mount an antibody response to the vaccine was the administration of immunosuppressive drugs, particularly mycophenolic acid (MPA) (2, 3, 7).

In a recent study,(7) we showed that only 35% of 120 RTR developed neutralizing antibodies (NA) to the BNT162b2 mRNA vaccine, compared to 97.5% of 202 immunocompetent controls. In addition, NA geometric mean titers (GMTs) in RTR were significantly lower than those in the healthy population. Following the second BNT162b2 mRNA vaccine dose, the vast majority of RTR thus remained unprotected and susceptible to infection, leading to high rate of morbidity and mortality from COVID-19 infection in the vaccinees (8, 9). SOT recipients had an 82-fold higher risk of breakthrough infection and 485-fold higher risk of breakthrough infection with associated hospitalization and death compared to the general population.(10).

In July 2021, the Israel Government approved administration of a third booster dose of the BNT162b2 mRNA vaccine for all SOT recipients and other immunocompromised patients. The BNT162b2 mRNA vaccine, which has been found effective against the B.1.617.2 (delta) variant that has now been detected across the globe (11), was the only vaccine administered across the population in Israel.

Given the diminished antibody response observed following the two doses of the BNT162b2 mRNA vaccine in RTR, we sought to analyze the receptor-binding domain (RBD) IgG and NA responses to an homologous booster dose of the BNT162b2 vaccine in our population of RTR, with the aim to reveal predictors for serologic response, focusing specifically on the prior response detected following the second vaccine dose. Our working hypothesis was that the humoral response elicited in RTR to a third BNT162b2 dose would be higher than the reported response following the second dose. We also monitored the adverse events (AE) subsequent to the booster dose in our population.

## Methods

### Study Population

This prospective study was conducted at the out-patient RTR clinic at Sheba Medical center. Ninety nine RTR who had previously received two doses of the BNT162b2 vaccine were vaccinated with an homologous third dose of the vaccine. Patients with a positive SARS-CoV-2 polymerase chain reaction test before or after the full two-dose vaccination were excluded from the study. Given the stronger response to the BNT162b2 vaccine in patients who received the vaccine prior to kidney transplant, patients vaccinated before transplant were also excluded. Vaccination was avoided during the first 3 months following transplantation and during active treatment for rejection. On the day of the third vaccination, blood was drawn, prior to administration of the booster dose, for baseline serology assessment of RBD IgG and NA. Three to 4 weeks following the booster dose, testing for RBD IgG and NA was repeated to assess the humoral response to the vaccine. For 76 of the 99 participants, we had RBD IgG levels 1 month post second vaccine. Written informed consent was obtained from all participants. The protocol and informed consent were approved by our Institutional Review Board (8314–21-SMC).

### Immunosuppression

As described previously (7), the standard maintenance immunosuppression regimen for our RTR patients is a calcineurin inhibitor (usually tacrolimus), an anti-metabolite, usually a mycophenolate-based drug (mainly MPA), and prednisone. An early steroid withdrawal protocol is implemented between the fifth and eighth days post-transplant for RTR with a low immunological risk for rejection. The two-drug maintenance regimen for these patients is usually comprised of tacrolimus and MPA. Conversion to a mammalian target of rapamycin (mTOR) inhibitor (sirolimus or everolimus) is instituted according to the patient’s risk of malignancy and intolerance to calcineurin inhibitors.

### Primary Outcome

A positive response to the third booster dose of the BNT162b2 vaccine was defined as RBD IgG ≥1.1 and the presence of NA capable of reducing viral replication by at least 50% at a 16 fold dilution or above.

### Data Extraction and Study Assessments

Patient information was obtained from the electronic patient records at the Sheba Medical Center, as described previously (7), and presented in Table 1. The MDClone data acquisition system at the Sheba Medical Center, which allows facile data retrieval, was used to retrieve average biochemical parameters that were recorded during 1 month prior to the third vaccine and any other relevant additional biochemical and clinical information (including average systolic and diastolic blood pressures in the 3 months prior to the booster dose, weight and BMI on the day of the third vaccine, average HbA1C level in the 6 months prior to the vaccine and total daily dose of the immunosuppressive medications on the day of the third vaccine, as described previously (7), and presented in the Table 2). In 15 patients, total daily mycophenolate dose was converted to the equivalent MPA dose by dividing the mycophenolate dose by 1.388. The use of cyclosporine, azathioprine, rapamycin and everolimus on the day of the third vaccine was also retrieved from the MDClone system.

**TABLE 1:** Demographic, clinical and biochemical characteristics of renal transplant recipients (RTR) stratified by antibody response.

Variable	Total cohort (N = 99)	Negative (N = 14)	Positive (N = 85)	p value
RTR characteristics				
Age, years, [median (IQR)]	66 (53–73)	71.5 (68–74)	63 (52–72)	**0.008** ^b^
Female sex, n (%)	25 (25.3)	4 (28.6)	21 (24.7)	0.76
Transplant to 3rd vaccine, years [median (IQR)]	3.4 (1.4–9.2)	2.8 (1.0–6.2)	3.6 (1.4–10.0)	0.25
2nd to 3rd vaccine, days [median (IQR)]	175 (171–178)	177.5 (174–178)	175 (170–178)	0.34
3rd vaccine to antibody testing, days [median (IQR)]	21 (21–21)	21 (21–33)	21 (21–21)	0.28
ESRD etiology, n (%)				
APCKD	14 (14.1)	0 (0)	14 (16.5)	0.15
Diabetic nephropathy	20 (20.2)	6 (42.9)	14 (16.5)	
Glomerulonephritis	28 (28.3)	3 (21.4)	25 (29.4)	
Nephrosclerosis	14 (14.1)	2 (14.3)	12 (14.1)	
Other	16 (16.2)	3 (21.4)	13 (15.3)	
Unknown	7 (0.1)	0 (0)	7 (8.2)	
ESRD secondary to DN	20 (20.2)	6 (42.9)	14 (16.5)	**0.02** ^a^
Time on dialysis, years [median (IQR)]	0.6 (0–1.5)	0.6 (0–3.0)	0.6 (0–1.5)	0.99
Transplant number, n (%)				
1	92 (92.9)	13 (92.9)	79 (92.9)	0.64
2	4 (4)	1 (7.1)	3 (3.5)	
3	3 (3)	0 (0)	3 (3.5)	
Donor type, n (%)				
Living	81 (81.8)	12 (85.7)	69 (81.2)	0.82
Deceased	16 (16.2)	2 (14.3)	14 (16.5)	
Unknown	2 (2)	0 (0)	2 (2.4)	
Medical history				
Hypertension	74 (74.7)	10 (71.4)	64 (74.1)	0.83
SBP 3-months average [median (IQR)]	131.8 (120.0–141.5)	139.5 (117.5–153.5)	131 (120.0–140.9)	0.25
DBP 3-months average [median (IQR)]	73.5 (68.0–79.5)	73 (66.8–79.5)	73.5 (68.0–79.5)	0.61
Ischemic heart disease	10 (10.1)	1 (7.1)	9 (10.6)	0.69
Congestive heart failure	10 (10.1)	2 (14.3)	8 (9.4)	0.58
Diabetes	37 (37.4)	7 (50)	30 (35.3)	0.29
HbA1C 6-months average (%) [median (IQR)]	6.4 (5.7–7.1)	6.4 (5.8–7.6)	6.4 (5.7–6.9)	0.59
Weight, (kg) [median (IQR)]	80 (70–92)	82.1 (70–89)	79.1 (70–92.2)	0.79
BMI, kg/m^2^ [median (IQR)]	26.9 (23.2–31.1)	26.6 (23.2–31.8)	27 (23.6–30.9)	0.91
Average Laboratory results 1 month before antibody testing day [median (IQR)]				
White blood cell (K/μL)	7.3 (6.1–8.9)	7.1 (6.4–8.1)	7.4 (6.0–9.0)	0.73
Lymphocyte absolute (K/μL)	1.7 (1.3–2.1)	1.6 (1.2–1.7)	1.7 (1.4–2.2)	0.18
Neutrophils absolute (K/μL)	4.6 (3.6–5.7)	4.3 (3.9–5.2)	4.8 (3.6–5.7)	0.99
Neutrophil/lymphocyte ratio	2.7 (2.0–3.5)	2.9 (2.6–3.2)	2.7 (2.0–3.6)	0.48
Hemoglobin (g/dl)	13.2 (12.2–14.0)	12.7 (11.8–13.4)	13.2 (12.3–14.0)	0.36
Platelets (K/μL)	179 (149–223.5)	175 (168–196)	182 (147.5–225.3)	0.91
Creatinine (mg/dl)	1.1 (0.9–1.4)	1.4 (1.2–1.7)	1.1 (0.9–1.3)	**0.03** ^a^
eGFR (CKD-EPI)**	64.7 (51.3–82.7)	46.6 (37.4–53.7)	67.9 (54.0–83.6)	**0.008** ^b^
Glucose (mg/dl)	115.5 (101–145.2)	129 (123–170)	113 (100.9–141)	0.057
Albumin (g/dl)	4.1 (3.8–4.2)	4 (3.7–4.1)	4.1 (3.9–4.2)	0.36
Globulins (g/dl)	2.6 (2.4–2.9)	2.5 (2.3–2.7)	2.7 (2.4–2.9)	0.08
C-reactive protein (mg/L)	3.26 (1.17–8.79)	2.7 (1.52–6.72)	3.29 (1.08–8.88)	0.66

Abbreviations: ADPKD, autosomal dominant polycystic kidney disease; DBP, diastolic blood pressure; eGFR, estimated glomerular filtration rate; ESRD, end stage renal disease, SBP, systolic blood pressure.

a <0.05.

b <0.01.

** eGFR, was calculated according to the following CKD-EPI, formula: eGFR, 141* min (Scr/k, 1)α * max (Scr/k, 1)-1.209 * 0.993Age * 1.018 * 1.159 (if black) (where Scr - standardized serum creatinine; k = 0.7 if female, 0.9 if male; *α* = −0.329 if female, −0.411 if male; min = the minimum of Scr/k of 1; max = the maximum of Scr/k or 1).

When p value is significant, below 0.05 or below 0.01 the values are bolded.

**TABLE 2:** RTR Immunosuppression Treatment on third vaccine Day Stratified by Antibody Response.

Immunosuppressive therapy	Total cohort (N = 99)	Negative (N = 14)	Positive (N = 85)	p value
Tacrolimus, n (%)	87 (87.9)	12 (85.7)	75 (88.2)	0.79
Tacrolimus daily dose (mg) on 3rd vaccine date [median (IQR)]	2 (1.5–3.0)	2.5 (1.5–3.0)	2 (2.0–3.0)	0.66
Tacrolimus daily dose (mg) per weight (kg) on 3rd vaccine day [median (IQR)]	0.03 (0.02–0.04)	0.03 (0.01–0.04)	0.03 (0.02–0.04)	0.74
Tacrolimus trough level 1M average before 3rd vaccine day (μg/L) [median (IQR)]	6.79 (5.6–7.7)	6.4 (5.8–7.6)	6.8 (5.4–7.9)	0.73
Mycophenolic acid (MPA), n (%)	79 (76.8)	13 (92.9)	63 (74.1)	0.12
MPA daily dose (mg) on 3rd vaccine date, [median (IQR)]	720 (360–720)	720 (360–720)	720 (0.0–720)	0.19
MPA daily dose (mg) per weight (kg) on 3rd vaccine date, [median (IQR)]	7.7 (3.6–9.5)	8.2 (5.1–10.3)	7.4 (0.0–9.2)	0.25
Prednisone, n (%)	74 (74.75)	10 (71.4)	64 (75.3)	0.76
Prednisone daily dose (mg) on 3rd vaccine date, [median (IQR)]	5.0 (0.0–5.0)	5.0 (0.0–5.0)	5.0 (3.0–5.0)	0.58
Prednisone daily dose (mg) per weight (kg) on 3rd vaccine date, [median (IQR)]	0.05 (0.00–0.07)	0.06 (0.00–0.06)	0.05 (0.03–0.07)	0.57
Immunosuppressive regimen				
Tacrolimus + MPA + prednisone, n (%)	45 (45.5)	7 (50)	38 (44.7)	0.71
Tacrolimus + MPA, n (%)	22 (22.2)	4 (28.6)	18 (21.2)	0.54
Tacrolimus + prednisone, n (%)	16 (16.2)	1 (7.1)	15 (17.6)	0.32
Cyclosporine + MPA + prednisone, n (%)	5 (5.1)	1 (7.1)	4 (4.7)	0.7
Tacrolimus + azathioprine, n (%)	2 (2.02)	0 (0)	2 (2.4)	0.56
Tacrolimus + azathioprine + prednisone, n (%)	2 (2.02)	0 (0)	2 (2.4)	0.56
mTORi (everolimus or sirolimus), n (%)	5 (5.1)	0 (0)	5 (5.9)	0.35

Abbreviations: MPA, mycophenolic acid; mTORi- mammalian target of rapamycin inhibitor.

Patients were instructed to report (using a specific questionnaire) any systemic (fever, fatigue, headache, myalgia, chills, nausea/vomiting, paresthesia) and local (pain, redness, or swelling at the injection site) reactions occurring within 30 days after third vaccine dose and were actively screened for any other systemic and local complaints.

### Antibody Detection Assays

Samples from vaccinated RTR were evaluated with an enzyme-linked immunosorbent assay (ELISA) that detects IgG antibodies against the RBD of SARS-CoV-2 as previously published (12, 13). A SARS-CoV-2 pseudo-virus (psSARS-2) neutralization assay (NA) was performed (14) using a propagation-competent vesicular stomatitis virus spike, which was kindly provided by Gert Zimmer, University of Bern, Switzerland.

### Statistical Analysis

Descriptive statistics were expressed as frequencies and percentages for categorical data, and means ± standard deviation (SD) or median with interquartile range (IQR) for continuous variables. All continuous variables were assessed for normality by the Kolmogorov–Smirnov test and log-transformed as appropriate. Differences in baseline characteristics between the groups were tested using Chi-square for the categorical variables or t-test for the continuous variables. To compare the humoral response before and after the third vaccine dose, a paired t-test and McNemar’s test were used.

Multivariable logistic regression analysis was used to identify factors associated with the vaccine-induced antibody response in the entire cohort. To analyze the association between antibody response and demographic, clinical and laboratory variables, a multivariable logistic regression analysis was constructed with a positive antibody response as the dependent variable, while adjusting for potential confounders. The variables used in the multivariate analysis were those with a p value <0.15 in the univariate analysis and those of clinical and biological relevance. Results are presented as odds ratio (OR), 95% confidence intervals (CI) and p-values. The correlation between IgG and log-transformed NA was analyzed using Spearman’s correlation by two-tailed parametric t-test means with 95% CIs.

All data analyses were performed with the SAS 9.4 software (Cary, NC, United States). Scatter plots of log-transformed IgG and NA were obtained using GraphPad Prism 5.0 (GraphPad Software, Inc., San Diego, CA). A p-value of less than 0.05 was considered as the cut-off for statistical significance.

## Results

### Cohort Characteristics

Our study cohort comprised 99 RTR who received a third homologous booster dose of the BNT162b2 mRNA vaccine. Median age was 66 years (IQR, 53–73); 74 (74.7%) were males; and median body mass index (BMI) was 26.9 kg/m^2^ (IQR, 23.2–31.1). Among the 99 RTR, for whom median time from transplant was 3.4 years, 81.1% had received a living donor transplant, and 69.7% had undergone pre-transplant dialysis, with median pre-transplant dialysis time being 0.6 years (IQR, 0–1.5). As shown in Table 1, 74.7%, 37.4%, 10.1% and 10.1% had a past medical history of hypertension, diabetes, ischemic heart disease, and congestive heart failure, respectively. 45.5% of the patients received the three-drug immunosuppression regimen of tacrolimus-MPA-prednisone, while 22.2% of the patients were treated only with tacrolimus and MPA (Table 2). Overall 93.4% of RTR were treated with a calcineurin inhibitor (87.9% with tacrolimus and 6.06% with cyclosporine), 76.8% with MPA, and 74.75% with prednisone.

Median time from the third vaccine to antibody testing was 21 days (IQR, 21–21). Ninety-four (94.95%) of the recipients had RBD IgG titers ≥1.1. Nine of the 94 recipients testing positive for RBD IgG nonetheless exhibited a low mean RBD IgG titer of 1.89 and did not develop NA; these patients were therefore considered as non-responders. Based on the two criteria—RBD IgG and NA—our RTR cohort included 85 patients (85.9%) in the positive response group (RBD IgG ≥1.1 and NA ≥ 16) and 14 (14.14%) in the negative response group (RBD IgG < 1.1 or NA < 16).

### Univariate Comparison of Positive vs. Negative Response Groups

RTR who responded to the booster dose were younger, with a median age of 63 years (IQR, 52–75), as opposed to 71.5 years (IQR, 68–74) in non-responders (*p* = 0.008). The rate of end stage renal disease (ESRD) secondary to diabetic nephropathy was significantly lower in the positive vs. the negative response groups (16.5% vs. 42.9%, respectively, *p* = 0.02). Average glucose blood levels in the month before the third vaccine was lower in the responders than in the non-responders, with a p value approaching significance (*p* = 0.057). Renal allograft function was significantly higher in the positive vs. the negative response group [median estimated glomerular filtration rate (eGFR) of 67.9 ml/min, IQR (54–83.6) and 46.6 ml/min, IQR (37.4–53.7), respectively, *p* = 0.008). For all other demographic, clinical and laboratory variables, the differences between the groups were not significant (Table 1).

A lower use of MPA was demonstrated for patients with a positive antibody response (74.1% for responders vs. 92.9% for non-responders, with a non-significant p value of 0.12). The total daily dose and daily dose per kg weight of tacrolimus, MPA and prednisone were not significantly different between the responders and the non-responders. The antibody responses were similar for the positive and negative response groups for the different immunosuppressive regimens administered, including the triple regimen containing MPA and double regimen of tacrolimus and prednisone (Table 2).

The differences in the humoral response between the positive and negative responders to the third vaccine dose is shown in Table 3 (which also shows the humoral response to the second vaccine and prior to the third vaccine).

**TABLE 3:** RBD IgG and NA prior to third vaccine and post third vaccine stratified by Antibody Response to third vaccine.

Variable	Total cohort (N = 99)	Negative (N = 14)	Positive (N = 85)	*p* value
Baseline immune status on 3rd vaccine day				
Positive RBD IgG and NA on 3rd vaccine day, n (%)	32 (32.3)	1 (7.1)	31 (36.5)	0.03*
Negative RBD IgG and NA on 3rd vaccine day, n (%)	67 (67.7)	13 (92.9)	54 (63.5)	0.03*
IgG-RBD GMT on 3rd vaccine day (95% CI)	0.79 (0.65–0.96)	0.34 (0.23–0.51)	0.91 (0.74–1.12)	0.0005**
NA GMT on 3rd vaccine day, (95% CI)	17.46 (12.38–24.62)	6.56 (3.12–13.80)	20.51 (14.1–29.85)	0.02*
Response to 3rd vaccine				
IgG-RBD GMT post 3rd vaccine day (95% CI)	3.08 (2.76–3.45)	1.28 (0.87–1.86)	3.57 (3.28–3.88)	<0.0001**
NA GMT post 3rd vaccine day (95% CI)	362.2 (220.7–594.6)	7.25 (2.42–21.71)	689.9 (456.3–1043)	<0.0001**

Abbreviations: CI, confidence intervals; GMT, geometric mean titer; NA, neutralizing antibodies; RBD, receptor-binding domain.

*<0.05, **<0.001.

When *p* value is significant, below 0.05 or below 0.01 the values are bolded.

### Response to the Second Vaccine Dose vs. the Third Booster Dose of the BNT162b2 mRNA Vaccine in RTR

**FIGURE 1:**
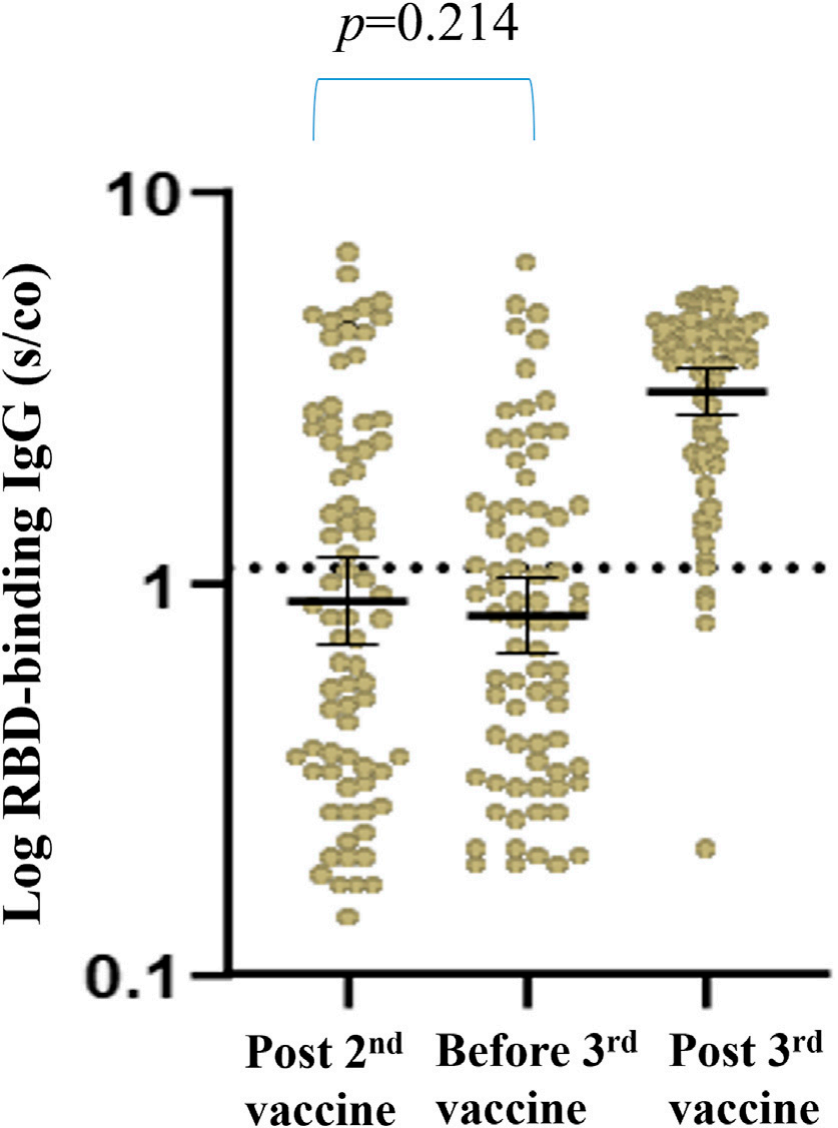
Geometrical Mean (GM) of RBD IgG Antibody levels post second vaccine, on third vaccine date and post third vaccine.

Of the 76 patients for whom RBD-IgG was assessed 1 month after the second vaccine dose [median of 25 days, IQR (18–42.5)], 32 (42.1%) had RBD IgG titers ≥1.1 with a GMT of 2.82 (95% CI, 2.35–3.39). At a median time of 175 days (IQR, 171–178) from the second vaccine, a third booster dose was administered, and all 99 RTR were tested for RBD IgG and NA immediately before the third vaccine dose was given. Based on the above two criteria (RBD-IgG and NA) for a positive vs. a negative response, 32 (32.3%) of the RTR had a positive response before the third vaccine, with a GMT for RBD IgG of 2.53 (95% CI, 2.07–3.11) and a NA GMT of 89.12 (95% CI, 53.03–149.8). The GMT for RBD IgG after the second vaccine dose was not significantly different from that observed before the third vaccine (Figure 1). Therefore we compared between the humoral response before and after the third dose in our total cohort of 99 RTR. The humoral response was assessed 3 weeks after the third booster dose [median time of 21 days, IQR (21–21)]. The positive response rate based on RBD IgG and NA titers had increased from 32.3% before the vaccine to 85.9% (85/99) after the third vaccine dose, with RBD IgG and NA GMTs of 3.57 (95% CI, 3.28–3.88) and 689.9 (95% CI, 456.3–1043), respectively. Both the rate and the intensity of response to the third booster dose were significantly higher than those observed before the booster dose (Table 4).

**TABLE 4:** Univariate Analysis for immune status before the third vaccine vs. post third vaccine in RTR.

	Before 3rd vaccine (N = 99)	Post 3rd vaccine (N = 99)	*p* value
All cohort			
IgG-RBD GMT (95% CI)	0.79 (0.65–0.96)	3.08 (2.76–3.45)	<0.0001**
NA GMT (95% CI)	17.46 (12.38–24.62)	362.2 (220.7–594.6)	<0.0001**
Positive responders			
n (%)	32 (32.3)	85 (85.9)	<0.0001**
IgG-RBD GMT (95% CI)	2.53 (2.07–3.11)	3.57 (3.28–3.88)	<0.0001**
NA GMT (95% CI)	89.12 (53.03–149.8)	689.9 (456.3–1043)	<0.0001**
Negative responders			
n (%)	67 (67.7)	14 (14.14)	<0.0001**
IgG-RBD GMT (95% CI)	0.45 (0.39–0.52)	1.28 (0.87–1.86)	0.001*
NA GMT (95% CI)	8.01 (5.92–10.84)	7.25 (2.42–21.71)	0.85

Abbreviations: CI, confidence intervals; GMT, geometric mean titer; NA, neutralizing antibodies; RBD, receptor-binding domain.

* <0.05, ** <0.001.

When *p* value is significant, below 0.05 or below 0.01 the values are bolded.

**FIGURE 2:**
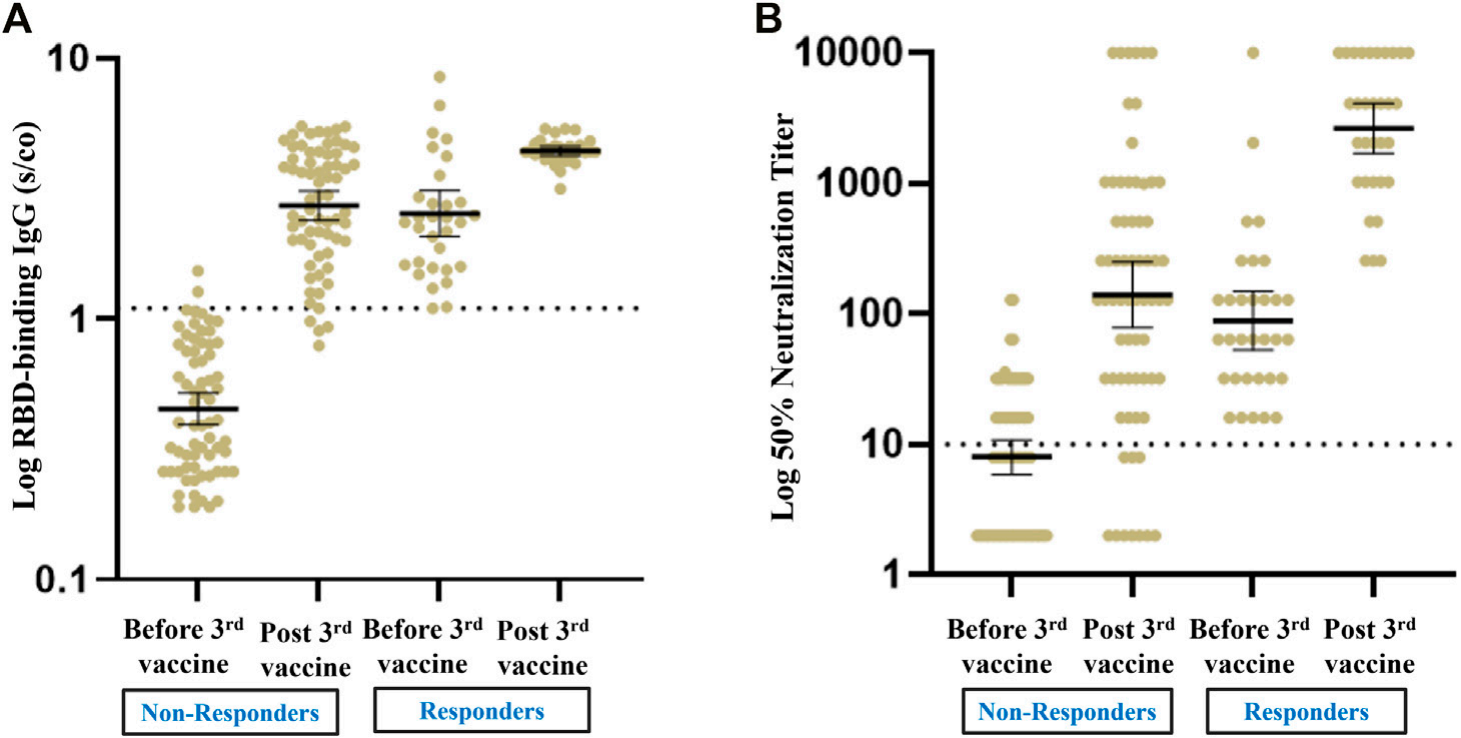
Antibody response pre and post third vaccine in RTR with a positive versus negative humoral response before the third vaccine. **(A)** Geometrical Mean (GM) of RBD IgG Antibody levels. **(B)** Neutralizing Antibody levels.

Of the 32 recipients with a positive humoral response prior to the third booster dose of the vaccine, 31 (96.9%) remained positive after the third vaccine, with a significant increase in GMTs for RBD IgG and NA. Sixty seven patients (67.7%) had a blunted antibody response before the third vaccine; among these, 54 (80.6%) exhibited a positive antibody response following the booster dose, with a significant increase in GMTs for RBD IgG NA (Figure 2).

### Multivariable Logistic Regression for Positive Antibody Response

Multivariable logistic regression analysis found that the likelihood for a positive response decreased by 94% in RTR above 65 years of age vs. below that age (OR = 0.06, 95% CI 0.00–0.88, *p* = 0.04). For every 1 ml/min increase in eGFR the odds for a positive response increased by 5% (OR = 1.05, 95% CI 1.00–1.09, *p* = 0.04). ESRD secondary to diabetic nephropathy was also found to be an independent predictor for antibody response (OR = 0.11, 95% CI 0.02–0.74, *p* = 0.02) (Table 5).

**TABLE 5:** Univariate and Multivariate Stepwise Logistic Regression Analysis for third vaccine Positive Antibody Response in RTR.

Effect	Univariate logistic regression		Stepwise logistic regression	
	Odds ratio	*p* value	Odds ratio (95% CI)	*p* value
	(95% CI)		
Age >65 vs. < 65	0.06 (0.01–0.47)	0.008	0.06 (0.00–0.88)	0.04*
Gender F vs. M	0.82 (0.23–2.89)	0.76	0.56 (0.07–4.52)	0.59
Time from transplant to 3rd vaccine, years	1.08 (0.95–1.23)	0.22	1.16 (0.96–1.4)	0.13
Time from 2nd to 3rd vaccine, days	0.98 (0.93–1.03)	0.43	1.01 (0.97–1.06)	0.55
Time from 3rd vaccine to antibody testing, days	0.94 (0.85–1.04)	0.22	1.02 (0.87–1.20)	0.81
ESRD secondary to DN yes/no	0.26 (0.08–0.88)	0.03	0.11 (0.02–0.74)	0.02*
eGFR (for every increase in 1 ml/min)	1.04 (1.01–1.08)	0.01	1.05 (1.00–1.09)	0.04*
Glucose per 1 mg/dl increase	0.99 (0.98–1.00)	0.14	…...............	…...............
Globulins per 1 mg/dl increase	5.56 (0.99–31.2)	0.05	7.56 (0.77–74.5)	0.08
MPA use yes/no	0.22 (0.03–1.78)	0.16	0.09 (0.01–1.12)	0.06

AbbreviationsDN, diabetic nephropathy; eGFR, estimated glomerular filtration rate; ESRD, end stage renal disease, MPA, mycophenolic acid.

a <0.05.

When *p* value is significant, below 0.05 or below 0.01 the values are bolded.

### Adverse Events

Adverse events were common, being recorded for 70.1% of the RTR cohort. Local and systemic adverse events were reported in 54.6% and 41.2% of the cohort, respectively. Pain at the injection site was the most frequent local adverse event, being experienced in 50 (51.5%) recipients following the third vaccine dose. Systemic adverse events, mainly fatigue, were reported for 31 (31.96%) of RTRs, with headache and myalgia being experienced only by the positive responders. Recipients with a positive humoral response following the third booster dose were more likely to experience systemic adverse events than non-responders (45.2% vs. 15.4% respectively, *p* = 0.04). No other differences in the prevalence of local or specific systemic adverse events were found between the responders and the non-responders (Table 6). No episodes of rejection were observed, and renal allograft function remained stable at a mean follow up of 60 days following the third vaccine dose. No allergic responses were documented.

**TABLE 6:** Local and Systemic Adverse Events (AEs) Reported after the third booster dose of BNT162b2 Vaccine Stratified by Antibody Response.

AEs	Total cohort	Negative	Positive	p value
	(N = 97)	(N = 13)	(N = 84)	
Local AEs, n (%)				
Pain at injection site	50 (51.5)	5 (38.5)	45 (53.6)	0.31
Swelling	9 (9.3)	2 (15.4)	7 (8.3)	0.41
Redness	10 (10.3)	1 (7.7)	9 (10.7)	0.74
Systemic AEs, n (%)				
Fever	4 (4.1)	0 (0)	4 (4.8)	0.42
Fatigue	31 (31.96)	2 (15.4)	29 (34.5)	0.17
Headache	17 (17.5)	0 (0)	17 (20.2)	0.07
Myalgia	17 (17.5)	0 (0)	17 (20.2)	0.07
Chills	3 (3.1)	1 (7.7)	2 (2.4)	0.3
Nausea/vomiting	3 (3.1)	0 (0)	3 (3.6)	0.49
Paresthesia	2 (2.1)	0 (0)	2 (2.4)	0.57
Any local AE, n (%)	53 (54.6)	6 (46.2)	47 (56)	0.51
Any sytemic AE, n (%)	40 (41.2)	2 (15.4)	38 (45.2)	0.04*
Any AE, n (%)	68 (70.1)	7 (53.6)	61 (72.6)	0.17

a <0.05.

When *p* value is significant, below 0.05 or below 0.01 the values are bolded.

## Discussion

The humoral response (both rate and intensity) to the third homologous booster dose of BNT162b2 vaccine was found to be significantly higher than that observed following the full two-dose vaccination and the baseline immune status prior to the third vaccine. RTR with a positive, as opposed to a negative antibody response were younger and were characterized by a lower prevalence of ESRD secondary to diabetic nephropathy, lower glucose in the 1 month prior to the vaccine, better renal allograft function, and a lower use of MPA. A multivariable model adjusted for age, sex, and times from transplant to the third vaccine dose, from second to third dose, and from third dose to serology assessment revealed that age, ESRD secondary to diabetic nephropathy, and renal allograft function are independent predictors for the humoral response to the third booster dose. The booster vaccination of RTR with the BNT162b2 vaccine was associated with a high rate of adverse events, with the most prevalent adverse event being pain at the injection site. The prevalence of systemic adverse events, mostly fatigue, but also fever, headache, myalgia, chills, nausea/vomiting, and paresthesia was higher in recipients with a positive (compared to a negative) antibody response.

The few studies on the humoral response to a booster vaccine dose in transplant recipients have reported conversion rates of 49–70%, as follows. Of 101 SOT recipients given three doses of the BNT162b2 vaccine, the response rate increased from 40% before the third dose to 68% 4 weeks after the third vaccine, but only 44% of seronegative patients seroconverted following the third dose (15). In 30 SOT recipients, antibody titers increased after the third dose in all the patients with low positive antibody titers after the first two doses but in only one quarter of patients (6/24) with negative antibody titers (16). A third dose of mRNA-1273 vaccine induced neutralizing antibody positivity in 60% of SOT recipients compared to only 25% of the placebo group (17). A study of the humoral response to a third dose of the mRNA-1273 SARS-CoV-2 vaccine in 159 RTR with a minimal response to the full vaccine showed that the overall response rate to the booster dose was 49%, with a higher response rate in those with a weak compared to a negative response following the second vaccine (81.3% vs. 27.4% respectively) (18). In 71 RTR homologously vaccinated with the BNT162b2 there was an increase in the serological conversion rate from about 50% after the second dose to about 70% 1 month after the third dose (19). In a recent study, 10 RTR who had failed to respond to a second dose of the BNT162b2 vaccine received a third dose of the mRNA-1273 vaccine, which induced humoral and cellular responses in 60% and 90% of the patients, respectively (20). By analyzing both antibody and neutralizing levels, we observed a strong response to the third, booster dose, with an increase in the positive response rate from 32% before the third dose to 85.9% thereafter. In addition, in our cohort the booster dose elicited a strong and effective humoral response in RTR who were either seropositive or seronegative before the administration of the booster: 80.6% of the recipients who had not responded to two doses of the vaccine became seropositive following the third dose, and the intensity of the humoral response largely improved in those who were seropositive prior to the vaccine (Figure 2). The differences between studies observed in humoral response following a third dose in RTR could stem from different characteristics of the cohorts as well as differences in sensitivity of testing assays used. Nevertheless, the advantages of a third dose to RTR are clear.

**FIGURE 3:**
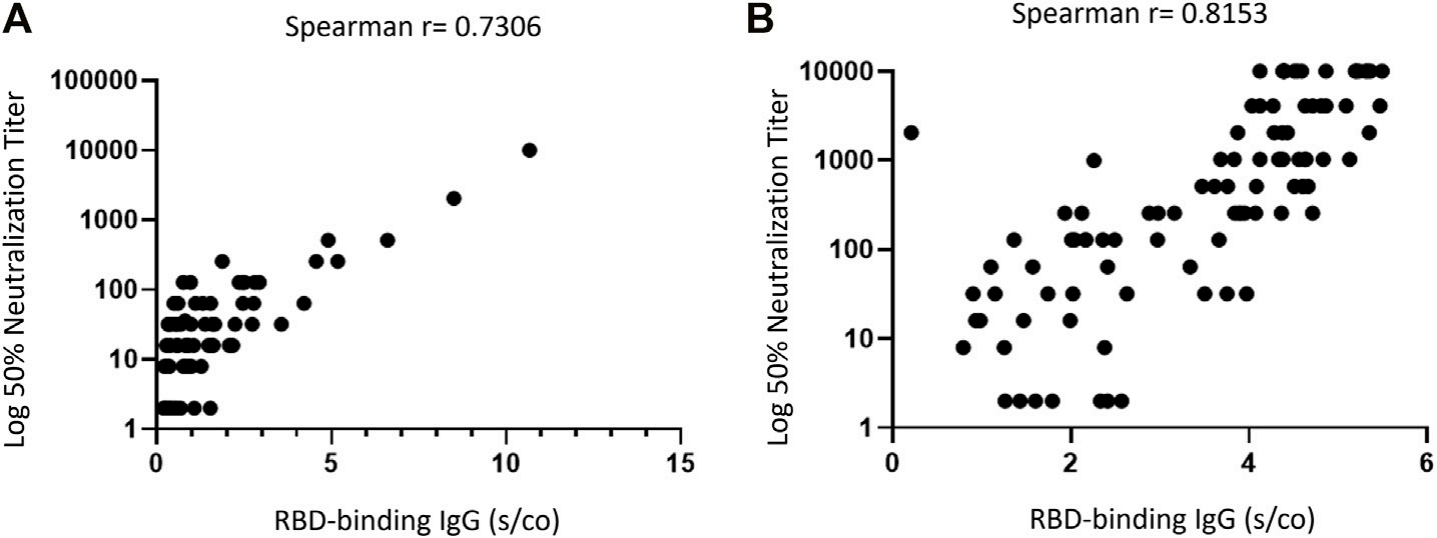
Correlation between RBD IgG and Neutralizing Antibodies in RTR. **(A)** On third vaccine date. **(B)** Post third vaccine. Each Dot Represents a Combined IgG-RBD and Neutralizing Antibodies Result for One Participant.

The importance of assessing NA is that they show an antibody functionality that encompasses both the quantity and the affinity of the IgG antibodies. The NA assay is considered the gold standard antibody assay for antibodies, and for SARS-CoV-2, it appears to be the *in-vitro* assay most closely correlated with protection (21). Indeed, a correlation between the level of NA to the SARS-CoV-2 spike protein and symptomatic disease was observed (22). Presence of NA to SARS-CoV-2 post natural infection has been shown to provide protection from asymptomatic and symptomatic reinfection (23). In addition, and despite the high correlation observed between RBD IgG and NA before and after the third vaccine dose (Figure 3), a substantial number of the RTR in our cohort with positive RBD IgG did not exhibit adequate neutralization activity and were therefore considered as negative responders (2% and 9.1% recipients before and after the third dose, respectively). The use of NA is therefore crucial in the assessment of the humoral response to reduce false positive results, which could make patients wrongly believe they are protected from the infection.

The robust response observed in our cohort following the third booster dose is not surprising, given prior data linking vaccination strategies with higher, additional and booster doses to superior immunogenicity responses in immunocompromised populations (24–28). Of note, although some types of immunosuppressive therapy, especially the use of MPA, were found to be major suppressors of the antibody response following the first and second vaccine doses, in our cohort MPA treatment did not significantly impact the ability to mount a humoral response after the third booster dose. MPA specifically blocks the proliferation of B and T lymphocytes via the inhibition of inosine-5-monophosphate dehydrogenase, thereby suppressing cell-mediated and humoral immune responses (29, 30). Despite the reduced antibody titers in RTR, cellular immune responses have been documented at considerable rate, even in seronegative vaccinated patients (4, 31). It is possible that for patients receiving immunosuppressive therapy antigenic re-exposure with a higher total antigen load, as achieved in natural infection, is needed to trigger and expand the reduced immune response to previous antigenic exposures.

In prior publications, older recipient age and a lower eGFR were associated with a negative response to the third booster dose (15, 19). Interestingly, we found that ESRD secondary to diabetic nephropathy is predictive for a blunted immune response to the third dose. This finding may probably be attributed to the direct effects of hyperglycemia and insulin resistance, causing an immune-compromised state in this population, as is manifested by dysregulation of both the innate and adaptive immune responses in people with diabetes (32, 33).

We found the third booster dose of the BNT162b2 mRNA vaccine to be safe. Although the prevalence of adverse effects was higher than that observed in our RTR following the first and second BNT162b2 doses (7), no serious adverse effects were reported. The high rate of adverse effects in our cohort, with an increased prevalence of systemic adverse effects in the positive responders, reflects an immune system activation post vaccine exposure in RTR capable of mounting an effective humoral response to the vaccine.

Certain limitations should be taken into consideration in interpreting our results. The study is not an efficacy trial (there is no control group), but NA have been demonstrated to have a significant correlation with protection from SARS-CoV-2. The implications of our findings are limited by the small number of patients and the short follow-up period after vaccination. Antibodies may wane over time, and the half-life of the neutralizing response cannot be predicted. Furthermore, cellular immunity was not assessed.

The above notwithstanding, our results are encouraging, given the high rate of seroconversion and the impressive response in previously seropositive patients. Based on our data, we believe that a third booster dose is essential for transplant recipients, irrespective of seronegativity/seropositivity prior to the vaccine, to achieve neutralization antibody activity and a higher degree of protection from COVID-19 infection. Despite the high response rate, it is likely that the booster vaccine-induced immunity is lower in RTR and other immunocompromised patients than in immunocompetent individuals. In a significant number of RTR, antibody titers following the third vaccine may be low or not associated with neutralization and protection. Therefore, we should not get caught up in complacency and keep searching for other strategies to improve patient protection. It is crucial that we continue to promote social distancing and masking as well as full vaccination of all transplant recipients, household members, and caregivers to provide a ring of protection for our immunocompromised patients.

## Data Availability

The raw data supporting the conclusion of this article will be made available upon reasonable request and not without undue reservation.

## Abbreviations

BMI, body mass index; CI, confidence interval; DN, diabetic nephropathy; eGFR, estimated glomerular filtration rate; ESRD, end stage renal disease; GMTs, geometric mean titers; MPA, mycophenolic acid; mTOR, mammalian target of rapamycin; NA, neutralizing antibodies; OR, odds ratio; RBD, receptor-binding domain; RTR, renal transplant recipients; SD, standard deviation; SOT, solid organ transplantation; IHD, ischemic heart disease; CHF, congestive heart failure; CVD, cardiovascular disease.
